# Supplementary material for: Abl family tyrosine kinases govern IgG extravasation in the skin in a murine pemphigus model
Source: Nat Commun. 2019 Sep 30;10:4432. doi: 10.1038/s41467-019-12232-3 (PMC6769004; doi:10.1038/s41467-019-12232-3)
Supplement: Supplementary file 1 — Supplementary figures [file 41467_2019_12232_MOESM1_ESM.pdf]

Supplementary information (Figures)

Abl family tyrosine kinases govern IgG extravasation in the skin in a murine pemphigus model

Ono et al

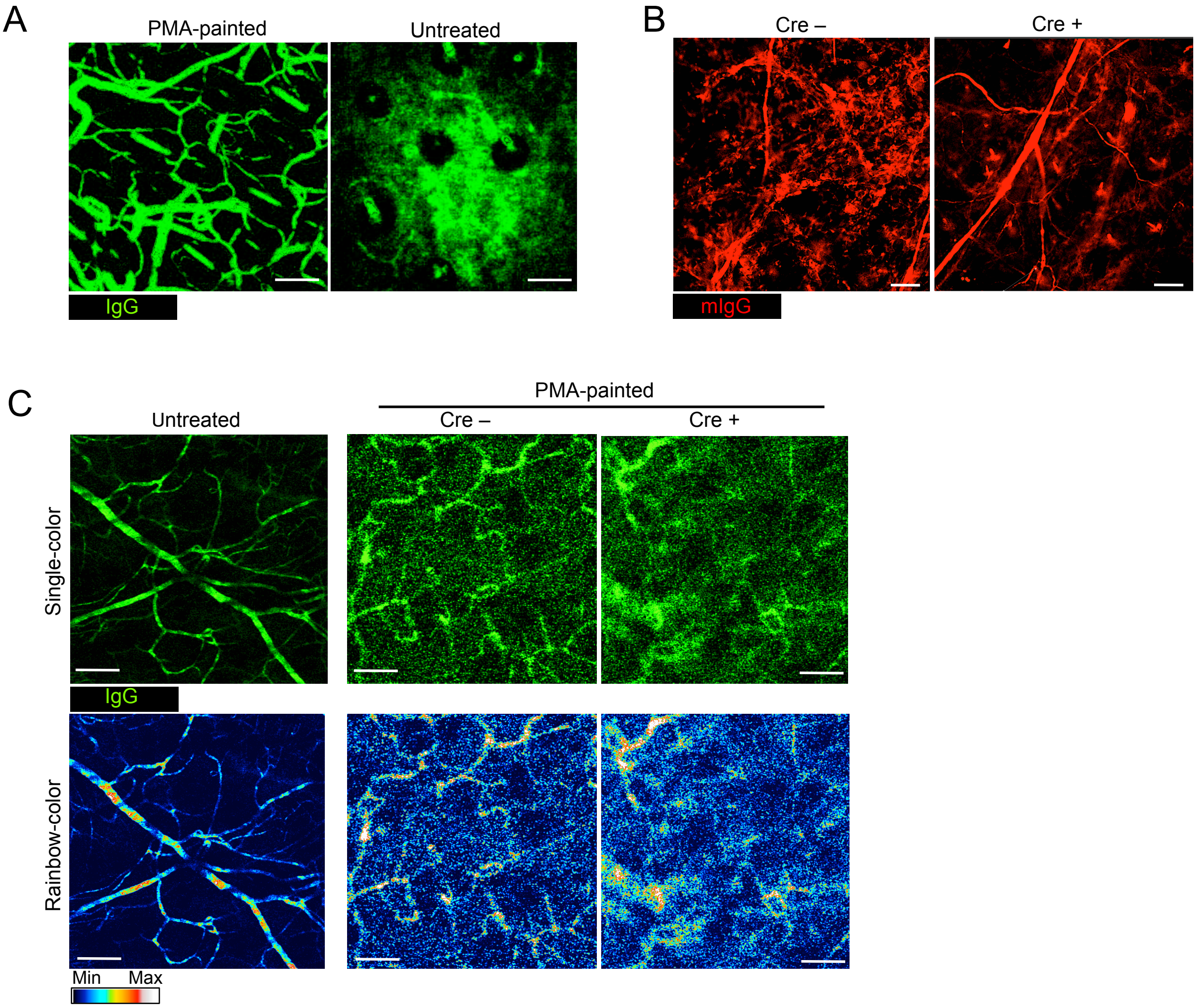

Supplementary Figure 1. IgG extravasation in the skin of VE-cadherin-CreERT2; D1D2-floxed mice

A, The single-color intensity scale of Fig. 2A, which shows time-lapse images of paracellular IgG leakage (15 min after intravenous injection of FITC-conjugated IgG via the tail vein) to the interstitium of ear dermis. Scale bar = 150 μm. **B,** Immunohistochemical images of ear skin dermis 24 h after intravenous injection of A594-conjugated mouse IgG. Scale bar = 100 μm. **C**, Time-lapse images of paracellular IgG leakage to the interstitium of the ear dermis of VE-cadherin-CreERT2; D1D2-floxed mice (15 min after intravenous injection of FITC-conjugated IgG via the tail vein). The single-color intensity images of FITC (upper panels) are converted to rainbow-color scale (lower panels) according to fluorescence intensity. Scale bar = 150 μm.

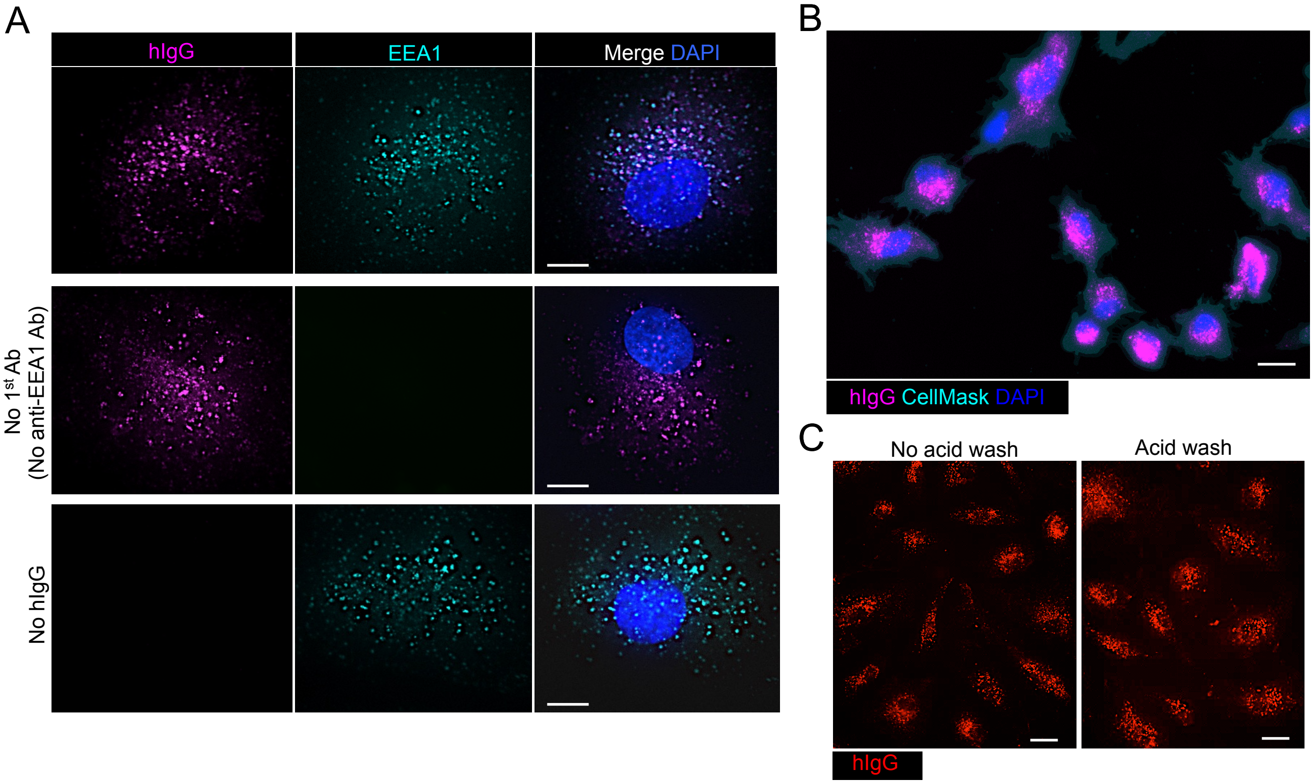

Supplementary Figure 2. Internalization of IgG in HDBECs.

A, Subcellular human IgG (hIgG) distribution in HDBECs (magenda), co-stained with anti-EEA1 Ab (cyan) (the upper panels). Blue represents a nucleus stained with DAPI. HDBECs stained without anti-EEA1 first Ab (the middle panels), and HDBECs without IgG endocytosis assay (the lower panels) were evaluated as negative controls for each staining. Scale bar = 10 µm.

B, HDBECs stained with CellMask Green Stain after IgG endocytosis assay. Scale bar = 20 µm.

C, HDBECs with or without acid wash after IgG endocytosis assay. Scale bar = 20 µm.

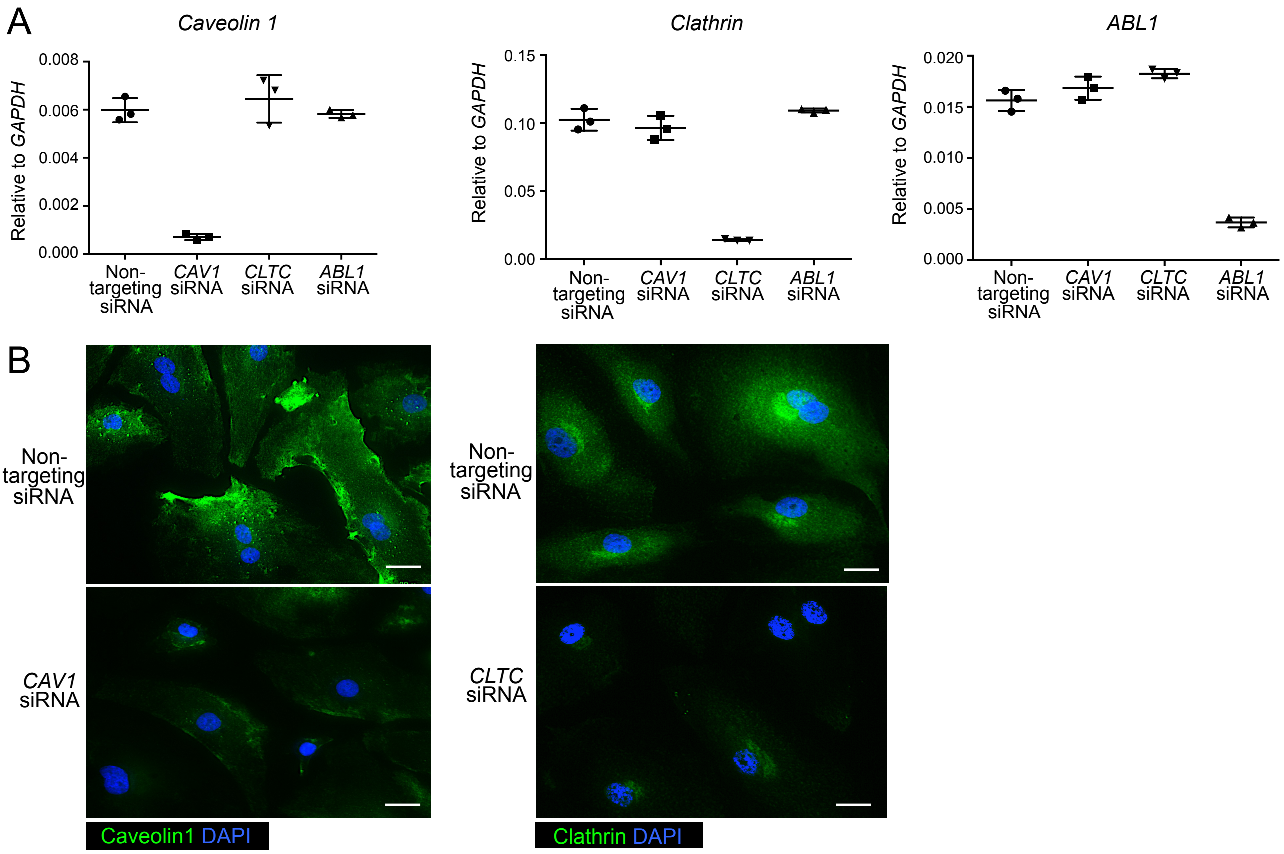

Supplementary Figure 3. The levels of mRNA or protein expression after each siRNA treatment.

A, The levels of mRNA expression after siRNA treatment against *caveolin 1* (*CAV1*), *clathrin* (*CLTC*), *c-ABL* (*ABL1*), or non-targeting siRNA (n = 3 wells, respectively). **B,** The levels of protein expression evaluated by immunocytochemistry of HDBECs after each siRNA treatment. Green represents caveolin 1 or clathrin. Blue represents nuclei stained with DAPI. Scale bar = 20 μm.

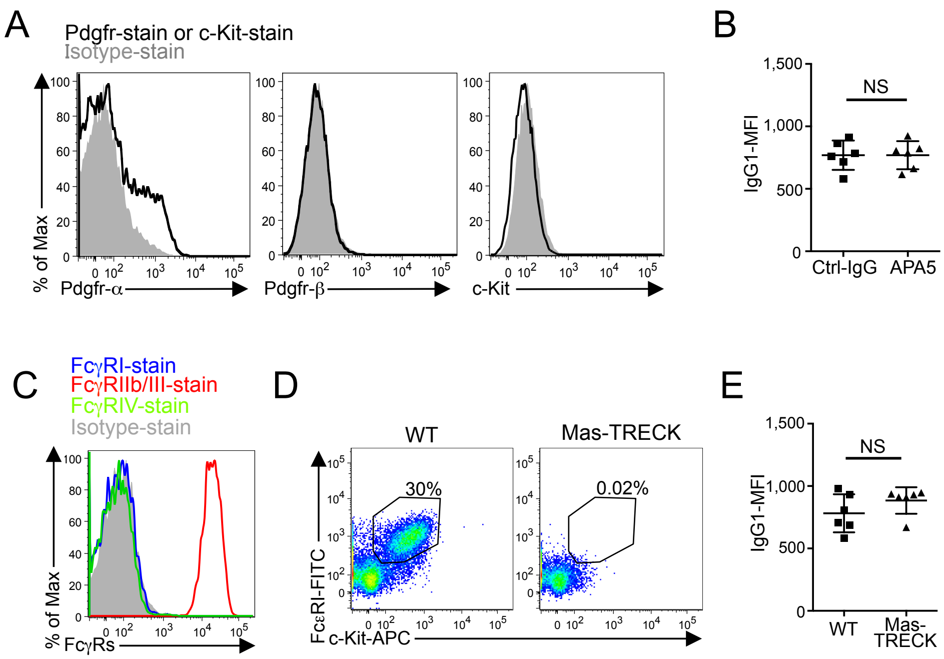

Supplementary Figure 4. Dispensable contribution of membrane-bound tyrosine kinases or perivascular mast cells for homeostatic IgG extravasation in the skin.

A, Expression of Pdgfr-α, Pdgfr-β, and c-Kit on BECs in mouse ear skin (solid line). Fill drawing represents isotype-stained control. **B**, IgG1-MFI of ear epidermis 24 h after AK18 injection pretreated with APA5 or control IgG (Ctrl-IgG) (n = 3, each. Both ears were evaluated separately). **C**, Expression of Fcγ receptors on mast cells in the ear skin (solid line). Fill drawing represents isotype-stained control. **D**, FcεRI^{+} c-Kit^{+} mast cells in wild type (WT) or Mas-TRECK mice after diphtheria toxin (DT) treatment. **E**, IgG1-MFI of ear epidermis in WT or Mas-TRECK mice 24 h after AK18 injection with DT treatment (n = 6, each).

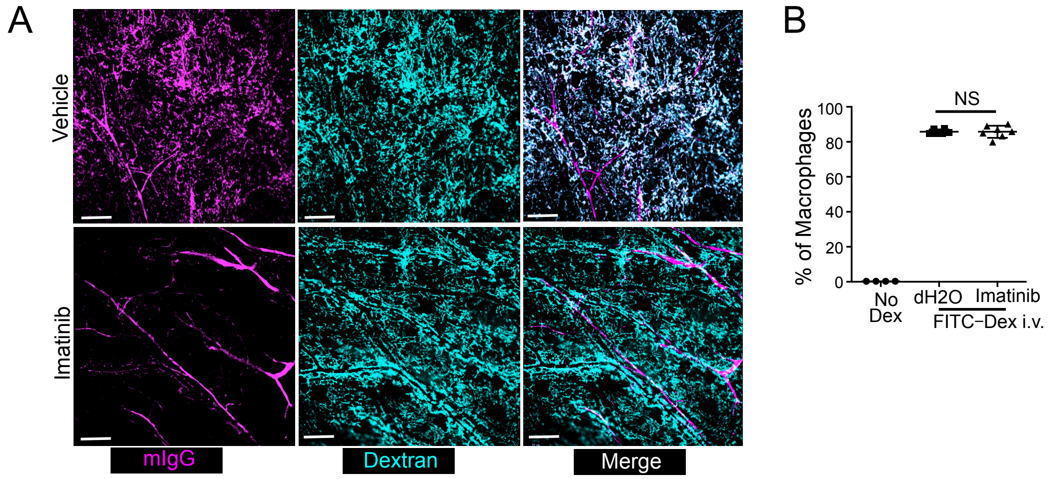

Supplementary Figure 5. Imatinib prevents extravasation of IgG but not of dextran in the skin.

A, Immunohistochemical images of ear skin dermis 24h after intravenous injection of A594-conjugated mouse IgG (magenda) and FITC-conjugated 150 kDa dextran (cyan), pretreated with imatinib or vehicle. Scale bar = 100 μm. **B**, The percentage of dermal macrophages positive for fluorescein after intravenous FITC-conjugated 150 kDa dextran with imatinib (6,000 μg $body^{-1}$) or vehicle pretreatment (n = 6 or 7, each), evaluated by flow cytometry. In each figure, the error bars represent the standard deviation of a data set.

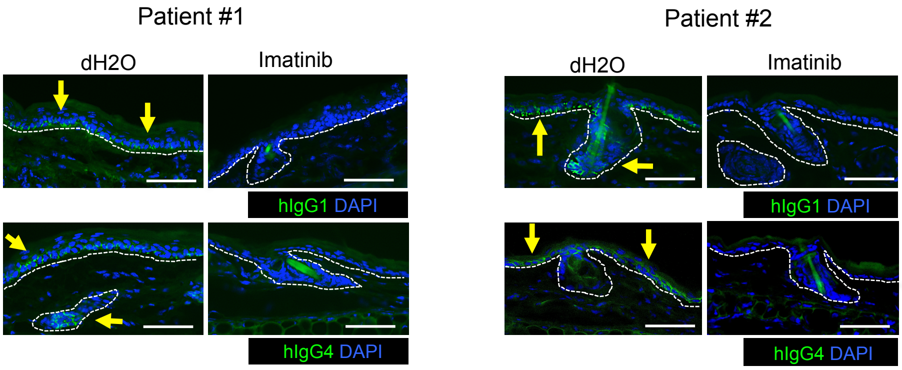

Supplementary Figure 6. Imatinib prevents extravasation of human pemphigus autoantibody in the murine skin.

Immunohistochemistry of anti-human IgG1 or IgG4 autoantibody deposition in the ears of mice pretreated with imatinib or vehicle, 24 h after intravenous injection of sera from two pemphigus patients. Yellow arrows represent epidermal Ab deposition. Blue represents nuclei stained with DAPI. Scale bar = 20 μm.

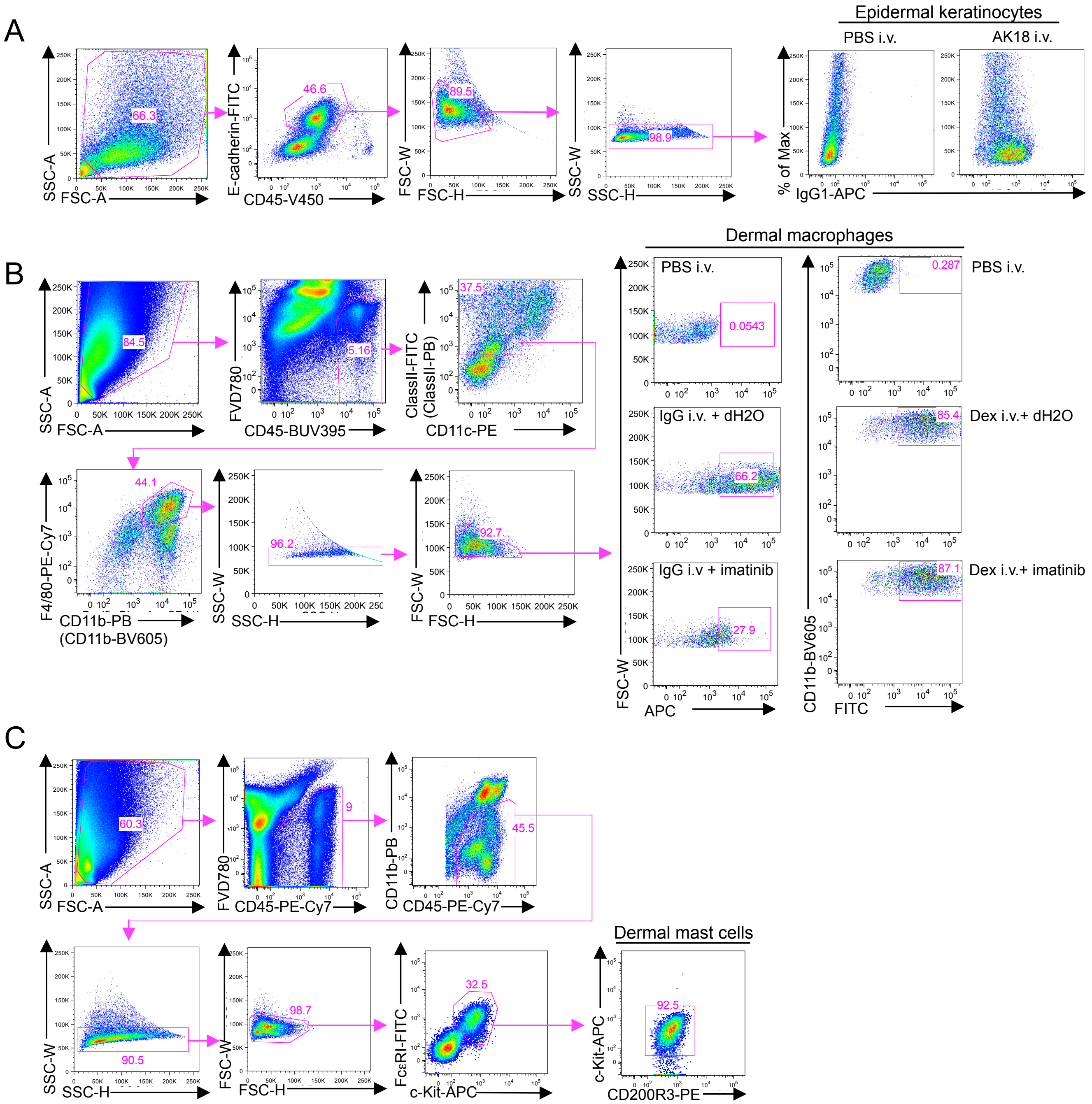

Supplementary Figure 7. Gating strategies for flow cytometric analyses.
A, Gating strategy to sort epidermis (CD45$^-$E-cadherin$^+$) cells from ears of mice for calculating epidermal IgG1-MFI, presented on Fig. 1B-D, 2E, 2H, 3F, 4B-I, and Supplementary Fig. 4B and 4E. **B**, Gating strategy to evaluate the percentages of dermal macrophages positive for A647-IgG, presented on Fig. 1G and 5H. When evaluating FITC-Dex in Supplementary Fig. 5B, MHC class II-PB and CD11b-BV605 were used instead. **C**, Gating strategy for mast cells, presented on Supplementary Fig. 4C-D.
